# Methionine Redox Homeostasis in Protein Quality Control

**DOI:** 10.3389/fmolb.2021.665492

**Published:** 2021-04-13

**Authors:** Laurent Aussel, Benjamin Ezraty

**Affiliations:** Aix-Marseille Université, CNRS, Laboratoire de Chimie Bactérienne, Institut de Microbiologie de la Méditerranée, Marseille, France

**Keywords:** methionine sulfoxide reductases, oxidized protein repair, HOCl, post-translational modification, oxidative stress

## Abstract

Bacteria live in different environments and are subject to a wide variety of fluctuating conditions. During evolution, they acquired sophisticated systems dedicated to maintaining protein structure and function, especially during oxidative stress. Under such conditions, methionine residues are converted into methionine sulfoxide (Met-O) which can alter protein function. In this review, we focus on the role in protein quality control of methionine sulfoxide reductases (Msr) which repair oxidatively protein-bound Met-O. We discuss our current understanding of the importance of Msr systems in rescuing protein function under oxidative stress and their ability to work in coordination with chaperone networks. Moreover, we highlight that bacterial chaperones, like GroEL or SurA, are also targeted by oxidative stress and under the surveillance of Msr. Therefore, integration of methionine redox homeostasis in protein quality control during oxidative stress gives a complete picture of this bacterial adaptive mechanism.

## Introduction

Environmental and cellular stresses can trigger perturbations in protein homeostasis, leading to misfolding and/or damage and making protein quality control an essential process in living cells. Protein oxidation generally results in structural modifications and can trigger aggregation, leading to loss of function that can impair cellular functions (Schramm et al., 2020). Oxidation can also result in protein degradation. However, chaperones contribute to protein protection and refolding, restoring their initial structure and/or biological functions. Interestingly, chaperones might act in concert with antioxidant enzymes dedicated to the repair of oxidized amino acyl residues. This combined action is poorly understood and little documented and will be the focus of the present review.

Within proteins, several amino acids can be oxidized by reactive oxygen species (ROS) and reactive chlorine species (RCS), the sulfur-containing amino acids cysteine (Cys) and methionine (Met) being particularly susceptible to oxidation. The rate at which HO° and hypochlorous acid (HOCl) react with these residues is rapid whereas it is slower with hydrogen peroxide (H_2_O_2_) (Buxton et al., 1988; Pattison and Davies, 2001; Davies, 2005). All living cells possess an intricate network of repair systems controlling the redox state of these residues that are highly prone to oxidation. Among them, methionine sulfoxide reductases (Msr) catalyze the reduction of methionine sulfoxide (Met-O) into methionine residues (Sourkes and Trano, 1953; Black et al., 1960). This activity places the Msr system at the heart of protein quality control. In *Escherichia coli*, the combined action of two enzymes, MsrA and MsrB, is required to repair the cytoplasmic protein-bound Met-O whereas only one enzyme, MsrP, deals with the periplasmic oxidized-proteins (Brot et al., 1981; Grimaud et al., 2001; Gennaris et al., 2015). This difference can be explained by the fact that, apart from some exceptions, Met residue oxidation relies on a racemic distribution in two diastereomers of R- and S-Met-O. Thereby, MsrA and MsrB exhibit a stereospecificity toward the S-form and the R-form, respectively, whereas MsrP reduces both isoforms (Brot et al., 1981; Grimaud et al., 2001; Gennaris et al., 2015). Another fundamental difference between MsrA/MsrB and MsrP relies on their catalytic mechanism. MsrA/MsrB catalyze a Cys-based redox mechanism involving a thioredoxin/thioredoxin reductase network (Boschi-Muller and Branlant, 2014) whereas MsrP relies on a molybdopterin-based reaction depending on the haem-containing membrane-bound MsrQ (Gennaris et al., 2015). Finally, a common feature arises from different studies reporting that bacterial strains lacking MsrA/MsrB or MsrP were affected in their virulence (Hassouni et al., 1999; Alamuri and Maier, 2006; Hitchcock et al., 2010; Mahawar et al., 2011; Singh et al., 2015). A more detailed description of Msr catalytic mechanisms and bacterial virulence can be found in a recent review (Ezraty et al., 2017).

Other oxidoreductases like fRMsr/MsrC, BisC, or TorZ/MtsZ specifically reduce free Met-O residues but are inactive toward protein-bound Met-O (Ezraty et al., 2005; Lin et al., 2007; Dhouib et al., 2016); they therefore can’t be part of the oxidized protein repair system. The DMSO reductase of *E. coli*, DmsA, has been reported to reduce Met-O residues mimicking substrates (Makukhin et al., 2019). Nevertheless, DmsA was not demonstrated to reduce Met-O within proteins. Recently, the *Rhodobacter sphaeroides* periplasmic DMSO reductase DorA-type has been elegantly shown to reduce protein-bound S-Met-O (Tarrago et al., 2020). In conclusion, MsrA, MsrB, MsrP, and DorA can reduce protein-bound Met-O residues and, *per se*, are involved in protein quality control processes.

In this review, we will present various and complementary approaches used to identify Msr targets and we will list bacterial proteins identified so far. We will also describe the combined effects played by chaperones and reductases in order to cope with oxidative stress and restore protein functions. Finally, we will highlight the Kafkaesque scenario where chaperones involved in protein quality control can be oxidized and inactivated by ROS and RCS, therefore becoming targets for the Msr system and conferring to the latter the role of ultimate sentinel in the cell.

## Bacterial MSR Substrates

Methionine is a hydrophobic residue containing an unbranched side chain with ample flexibility. This structural feature allows proteins containing Met-rich domains to interact with other partners. As Met-O is more hydrophilic than Met and exhibits an additional oxygen atom, its presence can modify the chemical environment as well as the steric hindrance inside proteins. Therefore, in most cases, oxidation of Met residues results in loss of protein structure and/or function. But this post-translational modification has also been reported to have a neutral or even a positive functional impact on the protein in a few cases. The high oxidation susceptibility of Met led to the concept that some Met residues might help to protect the rest of the protein from oxidation and act as an efficient endogenous antioxidant shield (Levine et al., 1996; Berlett and Levine, 2014). This feature of Met residues is now commonly called “The Stadtman theory.” Moreover, increasing evidence supports the idea that Met-O modifications can promote a transition from the inactive to the active form of a protein, acting as an on-off switch. The substitution of Met by glutamine (Gln), a mimetic of Met-O, is often used to test the functional consequences of oxidation of specific Met residues. This strategy was exploited to demonstrate the activation of HypT (hypochlorite-responsive transcription factor) through Met oxidation. Substitution of three Met residues to Gln resulted in a constitutively active HypT variant (Jo et al., 2019). Whatever the consequence on the protein activity (negative, positive, or neutral), the presence of Met-O in a polypeptide is a *bona fide* substrate for the Msr repair system. This latest family of proteins will be referred to as Msr substrates or Msr targets. It includes proteins which are under the surveillance of Msr for maintenance of their native structure and/or biological activity via Met redox homeostasis.

To identify this repertoire, many approaches have been used over recent decades. Bioinformatic analyses have been carried out to identify methionine-rich proteins in many bacterial proteomes. This enrichment can be global, leading to a high Met percentage (Met average = 2.9% in *E. coli*), or local, leading to Met hot spots in a short protein domain (Maisonneuve et al., 2009). Thereby, an *in silico* analysis of different proteomes allowed the *in vitro* identification of putative Msr substrates (Liang et al., 2012). Nevertheless, these candidates have to be confirmed by *in vivo* assays. Biochemical tests have been carried out on purified proteins, which were first subjected to different ROS or RCS *in vitro* (i.e., H_2_O_2_ or HOCl), and secondly repaired by Msr enzymes. Taking advantage of mass spectrometry analysis, the level of oxidation of each Met was measured, as well as the repair efficiency of Msr proteins. Historically, such experiments have been conducted with cytoplasmic substrates and repaired by MsrA and/or MsrB (Table 1). More recently, they have been carried out using periplasmic proteins and repaired by MsrP (Gennaris et al., 2015; Tarrago et al., 2018). Other tests rely on the comparison between a wild-type strain and *msr* mutants, different techniques being used such as two-dimensional electrophoresis or gel shift analysis as oxidized proteins exhibited a slower migration compared to their reduced form (Table 1; Liang et al., 2012; Ugarte et al., 2013; Gennaris et al., 2015).

**TABLE 1:** List of bacterial Msr substrates.

	Protein	Relevant function	Organism	*In vitro* evidence	*In vivo* evidence	Met% (*E. coli*)	Met rich domain*	References
**MsrA/MsrB cytoplasmic substrates**	AhpC	Alkyl hydroperoxide reductase	*H. pylori*	Protein activity Mass spec. analysis	Phenotypes	2.5		Benoit et al., 2013
	Ffh	Signal recognition particle (SRP)	*E. coli*	Enzymatic substrate/Protein activity/Mass spec. analysis	Phenotypes/Mass spec. analysis/Protein stability	5.9	√	Ezraty et al., 2004
	GlnA	Glutamine synthetase	*E. coli*	Peptide sequencing		3.4	√	Levine et al., 1996
	GroEL	Chaperone	*E. coli* and *H. pylori*	Protein activity/Mass spec. analysis/Co-IP		4 in *E. coli* and 3.4 in *H. pylori*	√	Khor et al., 2004; Alamuri and Maier, 2006
	Hsp 16.3	Membrane heat shock protein	*M. tuberculosis*	Mass spec. analysis/Gel shift assay/Protein activity		2		Abulimiti et al., 2003
	HypT	Hypochlorite-responsive transcription factor	*E. coli*	Mass spec. analysis/Protein activity	Phenotypes	1.65		Drazic et al., 2013
	KatA	Catalase	*H. pylori*	Co-IP/Mass spec. analysis	Protein activity	2.5	√	Alamuri and Maier, 2006; Mahawar et al., 2011
	L12	Ribosomal protein	*E. coli*	Enzymatic substrate		2.5		Brot and Weissbach, 1981
	MoeB	Molybdopterin-synthase sulfurylase	*E. coli*	Mass spec. analysis		3.6	√	Ezraty et al., 2005
	RecA	Recombinase A	*E. coli*	Protein activity Mass spec. analysis	Phenotypes/Gel shift assay	2.5	√	Henry et al., 2021
	SspB	Adhesins	*S. gordonii*	Mass spec. analysis/Gel shift assay	Phenotypes/Gel shift assay	0.66		Lei et al., 2011
	SSR	Site-specific recombinase	*H. pylori*	Co-IP		1.6		Alamuri and Maier, 2006
	UreG	Urease maturation	*H. pylori*	Protein interaction Protein activity Mass spec. analysis	Protein activity	4	√	Kuhns et al., 2013
**MsrP periplasmic substrates**	CysP	Thiosulfate- binding protein	*E. coli*	Mass spec. analysis		1.3		Gennaris et al., 2015
	DsbA	Thiol:disulfide interchange protein	*E. coli* and *R. sphaeroides*	Mass spec. analysis		3.2		Gennaris et al., 2015; Tarrago et al., 2018
	Ecotin	Inhibitor of pancreatic serine proteases	*E. coli*	Mass spec. analysis		2.8		Gennaris et al., 2015
	FecB	Fe^3+^ dicitrate-binding periplasmic protein	*E. coli*	Mass spec. analysis		2.5		Gennaris et al., 2015
	Ivy	Inhibitor of vertebrate lysozyme	*E. coli*	Mass spec. analysis		3.9	√	Gennaris et al., 2015
	LolA	Outer-membrane lipoprotein carrier protein	*E. coli*	Mass spec. analysis		1.1		Gennaris et al., 2015
	MalE	Maltose-binding periplasmic protein	*E. coli*	Mass spec. analysis		1.6		Gennaris et al., 2015
	MglB	D-galactose-binding periplasmic protein	*E. coli*	Mass spec. analysis		1.9		Gennaris et al., 2015
	MlaC	Probable phospholipid binding protein	*E. coli*	Mass spec. analysis		2.1	√	Gennaris et al., 2015
	MppA	Periplasmic murein peptide-binding protein	*E. coli*	Mass spec. analysis		1.4		Gennaris et al., 2015
	Pal	Peptidoglycan- associated lipoprotein	*E. coli*	Mass spec. analysis	Gel shift assay	3.9	√	Gennaris et al., 2015
	PotD	Spermidine/putrescine-binding periplasmic protein	*E. coli* and *R. sphaeroides*	Mass spec. analysis		2.8		Gennaris et al., 2015; Tarrago et al., 2018
	PpiA	Peptidyl-prolyl *cis-trans* isomerase A	*E. coli* and *R. sphaeroides*	Mass spec. analysis		2.4		Gennaris et al., 2015; Tarrago et al., 2018
	ProX	Glycine betaine-binding periplasmic protein	*E. coli* and *R. sphaeroides*	Mass spec. analysis		1.9		Gennaris et al., 2015; Tarrago et al., 2018
	PspE	Thiosulfate sulfurtransferase	*E. coli*	Mass spec. analysis		2.4		Gennaris et al., 2015
	RbsB	D-ribose-binding periplasmic protein	*E. coli*	Mass spec. analysis		1.5		Gennaris et al., 2015
	RcnB	Nickel/Cobalt homeostasis protein	*E. coli*	Mass spec. analysis		2.3		Gennaris et al., 2015
	SurA	Primary periplasmic chaperone	*E. coli* and *R. sphaeroides*	Mass spec. analysis	Phenotypes/Gel shift assay	3.4	√	Gennaris et al., 2015; Tarrago et al., 2018
	YmgD	Uncharacterized protein	*E. coli*	Mass spec. analysis		4.4		Gennaris et al., 2015
	ZnuA	High affinity Zinc uptake system protein	*E. coli*	Mass spec. analysis		2.1		Gennaris et al., 2015

*Met rich domain: 4 met/30 residues.

A crucial study was published in 2017 by Madeira and collaborators. Using a proteomic approach, the authors identified the Met-O content enrichment of the *Bacillus cereus* proteome in the *msrA msrB* mutant, giving an exhaustive view of the potential Msr substrates in this bacterium (Madeira et al., 2017). But the most convincing experiments in identifying Msr substrates have been the demonstration of the contribution of the Msr system *in vivo*. The inactivation of genes encoding the Msr system is predicted to exhibit a similar phenotype to the deletion of a gene encoding an Msr substrate. This observation was first made with the *E. coli* Signal Recognition Particle (SRP) as the *ffh* and the *msrA msrB* mutant strains were both affected in SRP-dependent protein export (Ezraty et al., 2004). Recently, the recombinase A (RecA) was found to be targeted by ROS and RCS, which converted four out of nine RecA Met residues to Met-O. The biological activity of the oxidized form of RecA was reported to be highly altered, but MsrA and MsrB were shown to reduce Met-O, restoring RecA catalytic activity *in vivo* and *in vitro* (Henry et al., 2021). Similar results were obtained with different substrates (AhpC, HypT, SspB) and in different bacteria (*Helicobacter pylori*, *Streptococcus gordonii*) (Table 1; Lei et al., 2011; Benoit et al., 2013; Drazic et al., 2013). Finally, a very important tool was put into place in 2019 with the publication of the MetOSite database^1^ which provides updated and manually curated data of sulfoxidation sites (Valverde et al., 2019). In early 2021, the database contained 7573 methionine sulfoxide sites found in 3701 different proteins identified in 30 species.

## Crosstalk of Chaperones and MSR Systems During HOCI Stress

Hypochlorous acid is the active ingredient of household bleach, but it can also be produced by neutrophils by the specific and abundant myeloperoxidase enzyme (Aratani, 2018). Its production is an efficient weapon against pathogens. HOCl is a strong oxidant which preferentially targets proteins and exhibits a high reactivity toward the sulfur-containing residues Cys and Met. Over the last decade, the Leichert, Jakob, and Collet groups have obtained important insight into the protection of bacterial proteins against aggregation during HOCl stress at the molecular level (Voth and Jakob, 2017; Goemans and Collet, 2019; Varatnitskaya et al., 2020). Extensive literature on this topic is available and in the following section, we will integrate Met-O reduction via the Msr system within the bacterial proteome protection network under HOCl stress. During this stress, proteins are oxidized and structurally modified, ultimately resulting in their aggregation. The ATP-dependent foldases GroEL/ES (Hsp60/Hsp10) and the DnaK/J/GrpE (Hsp70/Hsp40) systems simultaneously lose their activity via a drastic decrease in the cellular ATP amount and/or via their direct oxidation. To counterbalance foldase inactivation, bacteria rely on HOCl stress-induced ATP-independent holdases like Hsp33, RidA, and CnoX, which are activated either by oxidation or chlorination (Winter et al., 2005; Müller et al., 2014; Goemans et al., 2018). These chaperones prevent protein aggregation by binding unfolded proteins but have no protein refolding capacity. Moreover, inorganic polyphosphate (polyP) synthesized from ATP, acts as a chemical chaperone in a complementary way to the holdases (Xie and Jakob, 2019). Once the stress abates, holdases/polyP transfer their substrates to the GroEL/ES and DnaK/J/GrpE, which retrieve their activity in a scenario that operates like a well-oiled machine.

How the orchestration of the protection/refolding process with the redox control of the proteins occurs, including the reduction of Met-O by the Msr system, is still an open question. Work from the Maier group has provided some information as they identified in *Helicobacter pylori* a tripartite complex formed of KatA (a catalase as well as an Msr substrate), GroEL and MsrAB (MsrA and MsrB are fused in this organism) (Alamuri and Maier, 2006). Treatment of KatA with HOCl led to the oxidation of six Met residues, all of them being reduced by Msr *in vitro*. Nevertheless, no catalase activity has been recovered without the addition of GroEL to the MsrAB repair mixture (Mahawar et al., 2011). These results suggest that MsrAB and GroEL act in a cooperative manner to repair oxidatively damaged catalase and to maintain its enzymatic activity.

**FIGURE 1:**
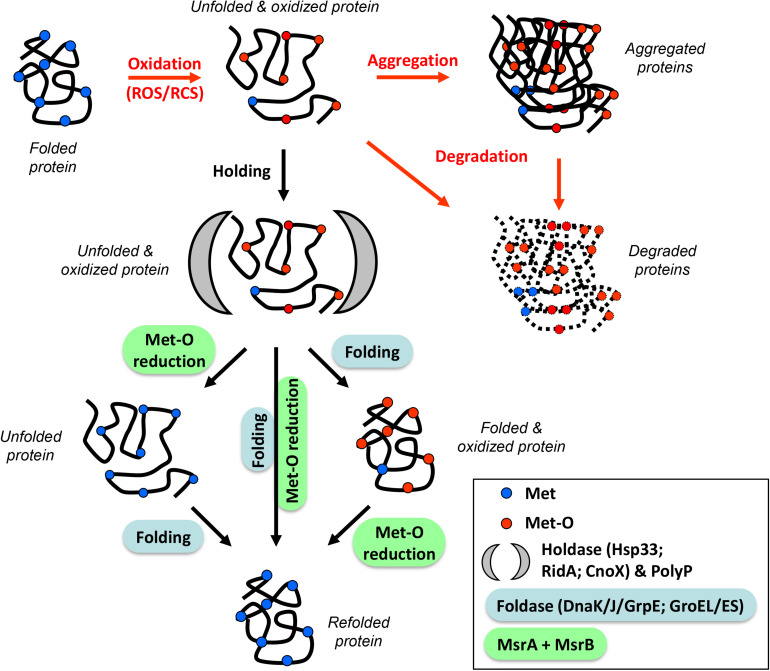
Orchestration of the protection/refolding process with methionine redox control. Reactive Oxygen/Chlorine Species (ROS/RCS) can oxidize proteins, leading to unfolded and oxidized molecules (containing Met-O residues). At this stage, proteins can aggregate and/or be degraded (red arrows). An alternative pathway involves the stress-induced holdase proteins (Hsp33, RidA, CnoX) or the chemical chaperone polyphosphate (black arrow) which prevent irreversible aggregation. After stress, these substrates are refolded by specialized foldases (DnaK/J/GrpE, GroEL/ES) and reduced by oxidoreductase (MsrA/B). Three possibilities are shown: (1) Met-O reduction followed by refolding (left), (2) refolding followed by Met-O reduction (right), and (3) simultaneous action of both systems (center). These three scenarios lead to a refolded and reduced protein.

To recover the reduced level and the tridimensional structure of a protein, three scenarios can be considered: (1) Met-O are first reduced into Met by the Msr system during or just after the holding step, and the ATP-dependent foldases then fold the protein to restore its initial structure, (2) ATP-dependent foldases act first in shaping the unfolded protein, followed by the reduction of Met-O residues into Met, and (3) both systems act simultaneously (Figure 1). It is tempting to rule out the third hypothesis due to a possible steric hindrance between the Msr enzymes and the chaperones. Nevertheless, MsrAB and GroEL were previously demonstrated to form a complex *in vivo* and to act in a cooperative manner *in vitro* (Alamuri and Maier, 2006; Mahawar et al., 2011), making this scenario possible. In 2012, Tarrago and Gladishev published an elegant article showing (i) that *in vitro* MsrA and MsrB were more efficient in reducing Met-O in unfolded than in folded proteins and (ii) that their activities increased with the unfolding state of their substrates (Tarrago et al., 2012). This increased activity was due to better access to oxidized Met in unfolded proteins. It also indicates that Msr serves a critical function in the folding process by repairing oxidatively damaged unfolded proteins. Thereby, the first scenario in which Met-O residues can first be reduced into Met before a final folding step catalyzed by ATP-dependent foldases might also be considered. However, all these hypotheses remain speculative as no specific study tackling this question has been carried out. *In vitro* protein repair with sequential addition of enzymes or interaction between holdases/foldases and the Msr enzymes could and should be considered in the future.

## The Kafkaesque Scenario: Molecular Chaperones Are Themselves Under MSR Surveillance

As previously mentioned, cellular housekeepers like GroEL or DnaK can be themselves targeted by ROS or RCS, leading to their inactivation and increasing their substrate’s susceptibility to oxidation and chlorination (Khor et al., 2004; Winter et al., 2005; Mahawar et al., 2011). Upon exposure of *E. coli* to HOCl or H_2_O_2_ combined with elevated temperature, DnaK loses its ability to protect proteins against aggregation (Winter et al., 2005). However, DnaK (15 Met/638 aa) was not shown to be protected from inactivation by the MsrA and MsrB enzymes. GroEL (23 Met/548 aa), another chaperone, is rather insensitive to H_2_O_2_ but efficiently modified and inactivated by HOCl (Khor et al., 2004). Under such conditions in *E. coli*, MsrA, and MsrB were demonstrated *in vitro* to restore a significant fraction of inactivated GroEL (Khor et al., 2004). In *Helicobacter pylori*, the MsrAB enzyme was shown to interact with the oxidized form of GroEL, this chaperone belonging to the repertoire of Msr substrates (Alamuri and Maier, 2006; Table 1). This interconnection between chaperone and Msr was also found in the periplasmic compartment. Indeed, the MsrP enzyme was shown to take care of the major periplasmic chaperone SurA (16 Met/428 aa), whose function is to escort β-barrel proteins to the outer membrane (Gennaris et al., 2015). *In vitro* HOCl-oxidized SurA loses its chaperone activity but this form can be repaired by MsrP, restoring the ability of SurA to protect unfolded substrates from aggregation. Moreover, remarkable *in vivo* evidence has been reported in monitoring for the first time oxidized protein repair by motility gel shift assay (Gennaris et al., 2015). All together, these results give Msr a central role in protein quality control homeostasis.

In conclusion, Msr enzymes are found in most living organisms, including species that are unlikely to encounter oxidants (in general) and HOCl (in particular) in their natural habitats. In the absence of exogenous stress, an open question remains on whether proteins exposed to low levels of ROS still need the Msr enzymes to maintain their biological activities. Therefore, a better understanding of the physiological importance of Msr during other types of stress will highlight the central role played by this ubiquitous oxidoreductase system. Future work will aim at integrating methionine redox homeostasis in protein quality control during oxidative stress to give a complete picture of this bacterial adaptative mechanism.

## Author Contributions

Both authors listed have made a substantial, direct and intellectual contribution to the work, and approved it for publication.

## Conflict of Interest

The authors declare that the research was conducted in the absence of any commercial or financial relationships that could be construed as a potential conflict of interest.

## Footnotes

1 metosite.uma.es
